# Potential Reasons for Unresponsiveness to Anti-PD1 Immunotherapy in Young Patients with Advanced Melanoma

**DOI:** 10.3390/life11121318

**Published:** 2021-11-30

**Authors:** Devayani Machiraju, Sarah Schäfer, Jessica C. Hassel

**Affiliations:** 1 Department of Dermatology and National Center for Tumor Diseases, University Hospital Heidelberg, 69120 Heidelberg, Germany; Devayani.Machiraju@med.uni-heidelberg.de; 2 Department of Dermatology, University Hospital Heidelberg, Ruprecht-Karls Universität Heidelberg, 69120 Heidelberg, Germany; Sarah.Schaefer@med.uni-heidelberg.de

**Keywords:** age, melanoma, immunotherapy, tumor antigenicity, Tregs, gender, gut microbiome, stress, radiation, vaccination

## Abstract

The impact of age on the clinical benefit of anti-PD1 immunotherapy in advanced melanoma patients has been evolving recently. Due to a reduced immune function in elderly patients, young patients with a robust immune system are theoretically expected to benefit more from the treatment approach. However, in contrast to this hypothesis, recent studies in patients with metastatic melanoma have demonstrated that immunotherapy, especially with anti-PD1 treatment, is less effective in patients below 65 years, on average, with significantly lower responses and reduced overall survival compared to patients above 65 years of age. Besides, data on young patients are even more sparse. Hence, in this review, we will focus on age-dependent differences in the previously described resistance mechanisms to the treatment and discuss the development of potential combination treatment strategies for enhancing the anti-tumor efficacy of anti-PD1 or PDL1 treatment in young melanoma patients.

## 1. Introduction

Compared to other cancers, the average age at diagnosis of melanoma among women is comparatively low at 60 years. Men develop the disease, on average, eight years later [1]. However, many patients diagnosed are even younger, and it is one of the most frequent types of cancer noticed in younger men and women aged less than 40 years [2,3,4]. Among the available systemic anti-cancer treatments for melanoma, immunotherapy with immune checkpoint inhibitors, such as anti-programmed cell death protein-1 (anti-PD1) or programmed cell death protein ligand-1 (PDL1), has demonstrated remarkable clinical benefits, increasing life expectancy in advanced disease patients [5,6,7,8]. Nevertheless, the clinical benefits are limited to a fraction of patients, and most patients do not respond to the treatment or eventually experience disease progression. Notably, when primary melanoma originates from the mucosal surfaces, the prognosis may be even worse [9,10].

Strikingly, the impact of age on anti-PD1 immunotherapy efficacy has been evolving recently. Due to immunosenescence in elderly patients, young patients with a robust immune system are expected to benefit from the treatment. In contrast to this hypothesis, studies in patients with metastatic melanoma have revealed a different picture (Table 1). In one of the first randomized clinical trials testing the PD-1 antibody nivolumab against the chemotherapy dacarbazine as first-line, a benefit from the PD-1 antibody compared to chemotherapy could be seen through all the age subgroups in a forest plot analysis; however, hazard ratios (HR) for overall survival (OS) decreased with increasing age (HR: 0.52 < 65 years, HR: 0.44 > 65 < 75 years and HR 0.25 > 75 years) demonstrating a possible higher benefit in older patients [11]. Similarly, in a multi-institutional retrospective study including 538 patients with advanced melanoma, the association between age and anti-PD1 treatment response was evaluated. The study showed that patients below the age of 62 were less likely to benefit from pembrolizumab treatment with a disease control rate of 52% compared to 63% in older patients [12]. Besides, the investigators noted that the likelihood of response increased with age, and observed that the odds ratios of progressing on pembrolizumab decreased 13% for every decade of patient age [12]. In the Danish national cohort, of 562 metastatic melanoma patients treated with pembrolizumab, 45% of patients who experienced disease progression were under 70 years, compared to 37% of patients above 70 years. A median OS of 18.8 months in patients under 70 years was reported compared with 36.5 months in patients above 70 years. Interestingly, this age-dependent difference in survival was not observed in patients treated with ipilimumab [13]. A report using the National cancer database of around twelve thousand advanced melanoma patients receiving immunotherapy with anti-cytotoxic T lymphocyte-associated protein 4 (anti-CTLA4) and/or anti-PD1 treatment showed an apparent decrease in OS of younger patients under the age of 60 compared to elderly patients of above 60 years. However, the number of patients who received anti-PD1 treatment is unclear in this study [14]. In addition, a recent meta-analysis including melanoma patients with anti-PD1 treatments from three prospective clinical trials further concluded that younger melanoma patients, under the age of 65, might experience a shorter OS compared to patients above the age of 65 [15]. Similar to previous reports, this study also found that age difference had little to no effect on OS in patients treated with anti-CTLA4 [15]. Although each study has its own limitation regarding the randomization or retrospective nature of the analysis, most of these trials have used 60–65 years of age as the point of intersection, and if any trend was noted, it was an HR indicating a better OS in the elderly patients treated with anti-PD1.

Both anti-CTLA4 and anti-PD1 revoke anti-tumor immune cells responses, but their timing and mechanism of action are different [20]. For a maximum clinical benefit from anti-PD1 treatment, pre-existing immune infiltration and, especially, tumor antigen-specific T cell reactivity in the tumor microenvironment (TME) is essential [21,22]. In brief, tumor cells trigger the cancer-immunity cycle by releasing antigens, T cells recognize those antigens presented by major histocompatibility complexes (MHCs) on antigen-presenting cells (APCs), and, subsequently, experience priming and activation [21,23]. Upon activation and proliferation, T cells travel to the tumor sites following a cytokine concentration gradient. When confronting the same antigen on MHCs, T cells release IFN-γ to enhance tumor killing. However, immune checkpoint proteins like PD1 and its ligand PDL1 are also needed to avoid the excessive activation of T cells and autoimmunity. Meanwhile, tumors take advantage of immune checkpoints to protect themselves from apoptosis induced by the immune system [24]. The release of IFN-γ from CD8+ T cells upregulates the expression of PDL1 on tumor cells. In parallel, TCR signaling upregulates the expression of PD1 on the T cell surface, which binds to PDL1 to exert adverse regulatory effects and blunt the anti-tumor function of T cells [25,26]. Anti-PD1 immunotherapy reactivates those T cells and significantly enhances anti-tumor effects in melanoma patients. However, the clinical data of anti-PD1/PDL1 therapy showed limited response rates, especially in younger patients. More disturbingly, the underlying mechanisms mostly remain unclear. 

**Figure 1:**
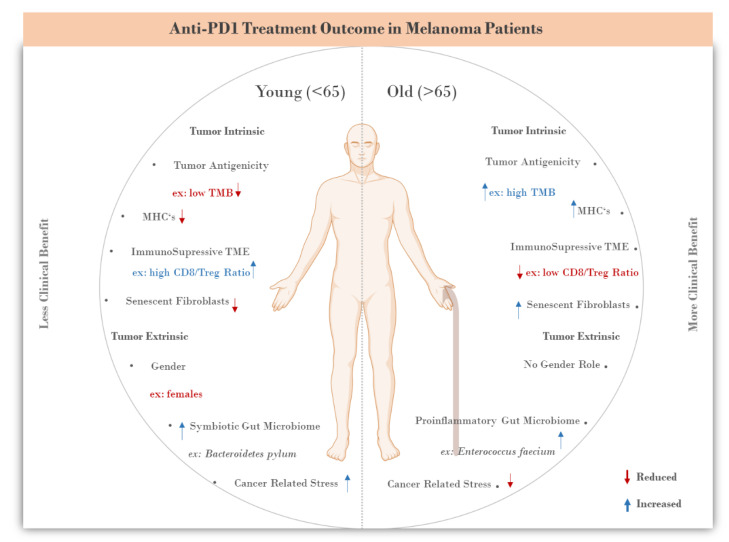
Age-related differences in clinical and molecular factors associated with anti-PD1 treatment outcome in metastatic melanoma patients.

Hence, in this review, we will focus on age-dependent differences in the previously described mechanisms of resistance to PD1/PDL1 inhibitors (Figure 1) and discuss the development of potential combination treatment strategies for enhancing the anti-tumor efficacy of anti-PD1/PDL1 treatment in younger melanoma patients.

## 2. Search Strategies and Selection Criteria

We searched Pubmed, MEDLINE, and references from relevant articles for prospective and retrospective clinical studies of anti-PD1 immune checkpoint inhibitors reporting the influence of age in melanoma patients. Keywords for literature search included melanoma, age, PD-1, PDL-1, nivolumab, pembrolizumab, and atezolizumab. All randomized clinical trials that had compared the efficacy of anti-PD1 as monotherapy in metastatic melanoma patients were selected. We excluded randomized clinical trials that compared the efficacy of anti-CTLA4 or anti-PD1/PDL1 in combination with anti-CTLA4. Only articles published in English are included.

## 3. Strong Immunoediting of Tumors in Young Patients 

Tumor Antigenicity: Effective anti-tumor immune responses require sufficient tumor antigenicity. Tumor cells produce abnormal proteins that distinguish them from their non-transformed counterparts. If the abnormal proteins on tumor cells are recognized by immune cells, they are likely to become neoantigens that facilitate the tumor cell recognition by T cells and the subsequent anti-tumor immune responses [27]. Tumor mutational burden (TMB) reflects the number of nonsynonymous mutations present in tumors and generates more neoantigens, hence, being more immunogenic [28]. In line with the theory, melanoma patients with high TMB in tumors respond better to anti-PD1 treatment [29,30]. Furthermore, mutations in DNA damage response and repair (DDR) genes usually lead to genomic instability, increase TMB, and enhance the anti-tumor immune response [31,32]. Several studies have revealed that DDR mutations are related to a better prognosis of advanced cancer patients receiving anti-PD1/PDL1 treatment [33,34]. Besides, it is essential to note that mutation quality is more important than quantity to induce potent anti-tumor immune responses. Accordingly, anti-PD1 treatment responses are shown to be more prominent when a clonal driver neoantigen is present in the tumors [35]. Although it is very evident that sufficient tumor antigenicity is required for a successful anti-PD1 treatment, so far, very few studies have been conducted to investigate the potential reasons for reduced efficacy to anti-PD1 treatment in the younger patients, mainly focusing on TMB and immunoediting mechanisms. 

For instance, molecular profiling of tumors in advanced melanoma patients receiving treatment with immune checkpoint inhibitors (ICIs) revealed that younger patients, under 65 years, have less TMB and neoantigens than patients above 65 years [36]. A recent study evaluating the influence of age on biomarkers to ICIs revealed that TMB significantly increased with patient age [37]. Similarly, in pan-cancer patients, TMB and the number of DDR pathway mutations were significantly lower in the younger group (<50) than those in patients aged above 50 years [38]. In addition, a recent study further revealed that younger patients accumulate driver mutations in their tumors that are less readily presented by their MHC molecules, indicating a more substantial toll by immune selection early in tumorigenesis [39]. Furthermore, some tumors may evolve to evade immune surveillance by losing the MHC molecules and upregulating immune checkpoint molecules on cell surfaces to regulate the magnitude and duration of T-cell responses [40,41]. Accordingly, a positive expression of MHC-II on tumor cells is associated with better therapeutic response and survival upon anti-PD1/PDL1 treatment in melanoma patients [42]. Conversely, reduced expression of immune-related genes such as HLA-I and HLA-II, which are responsible for presenting antigens to T cells, was observed in younger patients under 50 compared to those above 50 years of age [38]. 

Regulatory T cells (Tregs) in TME: both immunogenic tumor antigens and a functional immune system in TME are necessary to identify and kill tumor cells accurately and efficiently [22,43,44,45]. Among the T cell subsets, cytotoxic CD8+ T cells play a central role in anti-tumor immunity, whereas regulatory T cells (Tregs) contribute to the immunosuppressive capacity and dampen the anti-tumor immune response. Consequently, increased ratios of Tregs over CD8+ T cells within the TME is a significant factor that facilitates immune evasion and tumor growth [46,47]. In addition, increased infiltration of CD8+ T cells TME before anti-PD1 treatment indicates benefit from anti PD1 therapy [22,48]. In contrast, increased density of intratumoral follicular Treg cells exhibit a superior suppressive capacity and indicates reduced benefit to anti-PD1 treatment in the melanoma mice model [49]. Although PD-1 blockade is certainly a static method to revive exhausted anti-tumor CD8+ T-cells, on the other side, pre-existing Tregs present in the TME can also become more active and apply pronounced suppression on naive T cells undergoing activation [50]. In line with this, an increase in Treg frequencies was observed upon anti-PD1 treatment in leukapheresis specimens of non-responders in advanced melanoma patients [51], further indicating the resilience of Tregs in cancer is proving to be a prickle in anti-PD1 efficiency. 

Aging has a profound impact on our immune system. With increasing age, low-grade chronic inflammation (inflammaging) but reduced immune function (immunosenescence) is observed. Accumulation of effector memory T cells contributes to increased susceptibility to many aging-related chronic inflammatory diseases [52]. In addition, aging can also deteriorate Treg function, with Tregs from aged mice being less efficient than Tregs from young mice, which was proved to suppress conventional T cell function [53]. Besides, direct evidence of age differences in Tregs in TME was evolved [12]. Placing genetically identical tumors in aged mice significantly increased their response to anti-PD1 treatment compared with the same tumors placed in young mice. Interestingly, further analysis revealed that young mice had a significantly higher population of Tregs, skewing the ratio of cytotoxic and regulatory T cells in the TME. FOXP3 staining of human melanoma biopsies revealed similar increases in Tregs in young patients. The percentage of CD8+ T cells in melanoma TME was significantly lower in younger patients under the age of 66 years, which correlated with increased numbers in tumor-infiltrating FOXP3 regulatory T cells, indicating that tumors in young patients are accommodated with a more vital immunosuppressive environment with higher frequencies of potent Tregs. Hence, such differences in Tregs localization in TME may further account for the worse outcome to anti-PD-1 treatment in the younger patients compared to elderly patients [12]. 

Together, these data suggest that reduced efficacy of anti-PD1 treatment in the younger relative to the elderly group may be attributed to insufficient tumor antigenicity via reduced TMB, the unavailability of readily presentable tumor antigens, or even through the loss of MHC molecules, and increased frequencies of potent Tregs. At the same time, pro-inflammatory state and reduced Treg function in older people may provide unexpected age-favored clinical benefits to anti-PD1 treatment. Collectively, these data suggest that tumors developing in younger patients may be prone to stronger immunoediting than those in elderly patients. Hence, developing new combinational treatment strategies to counter immunoediting mechanisms of tumors such as increasing tumor antigenicity, and depleting Tregs in TME may improve anti-PD1 efficacy in younger patients and therefore needs to be further investigated.

## 4. Cancer-Associated Fibroblasts (CAFs) in TME

Among all the stromal cells that populate TME, fibroblasts are the most common elements and are critically involved in tumor progression. They regulate tissue architecture via extracellular matrix (ECM) and participate in the inflammatory response at the tumor site [54]. Aging has a profound influence on fibroblasts. As we age, fibroblasts go through a non-replicating (senescent) state and acquire senescence-associated secretory phenotype (SSPs) [55,56]. SASP induction in stromal populations leads to the persistently increased secretion of multiple inflammatory cytokines that maintains a low-grade adaptive immune response in older patients [57,58]. Besides, the secretome of aged fibroblasts releases increased amounts of secreted frizzled-related protein 2 (SFRP2) into the TME, which previously have shown to increase PD1 expression on T cells [59,60]. Accordingly, PD1 expression was shown to increase with age and contribute to the age-dependent functional decline in effector memory T cells [61]. The adaptive immune responses suppressed by senescent CAFs in TME may contribute to the anti-PD1 treatment response observed in older patients. Hence, an increased presence of senescent CAFs in TME may indicate a better clinical outcome. However, the continuing challenge for the development of a standardized panel of markers to identify senescent CAFs in TME hampers the approach and further research in this field may aid in identifying biomarkers.

## 5. Role of Female Gender in Young Patients’ Response to Anti-PD1 Treatment

Especially at a younger age (<50 years), melanoma incidence is higher in females than in males [2]. After years of study, it is clear that women generally mount a stronger immune response than men. Sex hormones and sex-chromosome-related genes are the main factors driving these differences in immunity [62,63]. In contrast to the expectations that females with a much stronger immune system may experience more clinical benefits to the anti-PD1 treatment, recent meta-analyses of clinical trials across cancer types treated with ICB indicate that young and female patients demonstrate low response rates, especially in melanoma [12,64,65,66,67]. Six out of seven clinical trials of melanoma, included in the meta-analysis, showed a clear OS advantage in male patients who received ICIs [67]. Furthermore, in this meta-analysis, among the six clinical studies that revealed an association between reduced overall survival and female gender, four studies predominantly involved anti-PD1 treatment as immunotherapy. In advanced melanoma patients who received immunotherapy majorly with anti-PD1 treatment, particularly young and female melanoma patients showed a reduced immunotherapy response in a retrospective study from a single center [68]. 

The molecular mechanisms underlying sex-based differences in non-responsiveness to ICIs have been recently characterized in mouse models, such as estrogen-mediated recruitment of myeloid-derived suppressive cells (MDSCs) and Tregs to the TME, that are known to be involved in resistance to ICIs [69]. Interestingly, such differences disappeared in ovariectomized mice when reconstituted by estradiol supplementation and modulated by tamoxifen [69]. In the same model, Estrogen-based modulation of the PD-1/PD-L1 pathway in FOXP3+Tregs cells was previously reported [70,71]. In addition, male tumors are more antigenic than female tumors. In a pan-cancer analysis, male-derived tumors displayed a higher density of somatic-coding single nucleotide variants (SNVs) than female-derived tumors [72]. In melanoma, more missense mutations are observed in men than women [73], and female patient tumors had less TMB when compared to male patient tumors. Female patients whose tumors had high TMB had better overall survival following immune checkpoint inhibition than female patients with low TMB [74]. In addition to the TMB, other cancer-associated germline antigens also show sex differences, with males expressing more cancer germline antigens than females [67]. The reduced antigenicity may further contribute to the compromised anti-PD1 treatment efficacy observed in female patients. Strikingly, a recent study revealed that young and female patients accumulate driver mutations in their tumors that are less readily presented by their MHC molecules [39], further suggesting that these effects are strong and complementary. Together, current knowledge provides a rationale for the paradigm that immune selection exerts its toll differently concerning age and gender, with a strong immunoediting effect being observed in younger and female patients. Prospective clinical trials stratified by age and gender in the randomization process may significantly add to a deeper knowledge of the role of gender in the anti-tumor activity of anti-PD1. A better understanding of the molecular mechanisms involved in the tumor immune escape would also help identify biomarkers of resistance to ICIs, differently expressed in young women.

## 6. Differences in Gut Microbiome in Young verus Elderly Patients

The gut microbiome is a principal factor in determining the host immune system response [75]. Mechanistic and reverse translational evidence in germ-free mice that lack intestinal microbiota revealed that different groups of bacteria influence distinct immune-modulating actions [76]. Interestingly, the diversity and composition of the gut microbiome were shown to influence response to anti-PD1 treatment in cancer patients [76,77,78,79,80]. In melanoma patients receiving PD-1 inhibitors, a lower alpha-diversity of the gut microbiome and relative abundance of *Bacteroidetes* phyla was associated with shorter survival and resistance to treatment [76], whereas, pre-treatment stool samples from 42 patients with metastatic melanoma receiving anti-PD1 therapy showed more commensals such as *Bifidobacterium longum*, *Collinsella aerofaciens*, and *Enterococcus faecium* species in responders than in non-responders [81]. It has to be noted that a shared pathway among these commensals is dendritic cell activation, induction of CD4+ and CD8+ T-cells, increased pro-inflammatory Th17, and associated interleukins (e.g., IL-17, IL-12), as well a decrease in IL-10 and Tregs [76,77,78,79,80,81,82,83,84]. 

The gut microbiota composition is dynamic over a lifetime and substantially differs between young and elderly hosts [85,86]. In general, young people predominantly have more abundant symbionts such as *Firmicutes* and *Bacteroidetes*. In contrast, there is a shift in older people towards more pro-inflammatory commensals such as proteobacteria [85,87]. Although the direct link between gut microbiome in young melanoma patients and reduced anti-PD1 efficacy, in particular, remains unclear, it can be observed that the members of the *Bacteroides fragilis, Clostridium* strains that are more prevalent in younger people control systemic inflammation by inducing FOXP3-positive Treg differentiation, production of interleukin-10 and transforming growth factor β [88,89]. Microbiome-derived-lipopolysaccharides (LPS) may play essential roles in immune modulation. For example, Bacteroidetes species may contribute up to 79% antagonistic forms of the LPS (A-LPS), which are involved in immune silencing [88,90]. Accordingly, Polysaccharide A (PSA) derived from the Bacteroides fragilis rebalances skewed systemic T helper responses and promoted Tregs by inducing human CD4+FOXP3+ T cells and enhanced suppressive function of circulating FOXP3+ in vitro [79]. Compared to young people, the gut microbiome in older people contains more pro-inflammatory bacteria that increase LPS levels (P-LPS) [91], which is best known for eliciting strong immune responses in humans [92]. The leakage of P-LPS and other microbial products from the gut upregulates cytokines such as interferons and interleukin-1 in circulation, and induces a pro-inflammatory state [78,93,94]. This might partially explain the age-favored clinical benefit of anti-PD1 treatment in older people as proteobacteria-derived LPS traditionally may drive immune infiltration to the TME, which may enhance treatment efficacy. An example of how bacteria may influence TME comes from a recent study showing that Pseudomonas aeruginosa LPS in mice with lung cancer enhances inflammatory cell recruitment throughout the tumor and induces PD1/PDL1 expression which leads to efficient anti-PD1 responses [95].

However, so far, there is no direct evidence reported of age-related differences in the gut microbiome of melanoma patients receiving anti-PD1 treatment. Nevertheless, based on similar tumor features in young patients and patients with unfavorable gut microbiome in non-responders to anti-PD1 treatment is noteworthy to study such differences. This may lead to a simple and supple solution to alter the gut microbiome in younger patients for maximum clinical benefits with anti-PD1 treatment.

## 7. Obesity and Response to Anti-PD1 Treatment

Aging is related to significant changes in body structure and increased abdominal obesity [96]. Both obesity and aging are marked by low-grade inflammatory state and endocrine changes. Interestingly, a positive role for obesity in ICI treatment has been recently reviewed [97]. Anti-PD1 or PDL1 led to significantly enhanced PFS and OS in overweight melanoma patients compared to normal-weight patients [98,99,100]. Obesity can alter nutrient availability in the TME and cause immune dysfunction by enhancing transcriptional and metabolic reprogramming events [101]. Furthermore, circulating T-cells have increased expression of PD-1 in diet-induced obese mice compared to control mice. Both CD4+ and CD8+ T-cells, when stimulated ex vivo, displayed a reduced ability to proliferate and produce cytokines compared to T-cells from normal-weight mice. Besides, increased leptin (adipokine) levels in obese mice have mediated the PD1 expression and T cell exhausted phenotype [102]. Meanwhile, obesity also impacts gut microbiome diversity [103] which can play a role in response to anti-PD1 efficacy in melanoma, as described in the previous section. Hence, suggesting that obesity-driven changes in peripheral and tumoral immune cells may facilitate the anti-PD1 therapy efficacy. Nevertheless, it is important to note that obesity is a common condition across ages; hence, it may not necessarily be an age-related factor associated with better outcomes to anti-PD1 treatment.

## 8. More Psychological Distress in Younger Patients 

Younger age is consistently associated with higher rates of physiological distress in patients with cancer [104]. Multiple responsibilities are inherent in midlife, maybe one of the reasons behind the stress. The cancer-specific distress may not fall into the classic description of anxiety or depression but is still disruptive to quality of life. The neuroendocrine factors, including stress hormones such as Glucocorticoids that increase in the context of psychological distress, are known to impact the immune system profoundly [105,106,107]. Corticosteroids impair activation of T lymphocytes by blocking T helper and recruiting T regulatory cells and can also induce M2 macrophages polarization [107]. Thus, stress-induced endogenous glucocorticoids may mediate potent immunosuppressive effects in young cancer patients. The molecular mechanism through which mental stress causes cancer therapy-relevant immunosuppression was observed by comparing social defeat in pre-conditioned and non-stressed control mice. This study revealed that mental stress caused a state of both local (within tumor microenvironment) and systemic immune suppression, as reflected by the inhibition of IFN responses, antigen signaling, chemotaxis of neutrophils, and myeloid leukocyte differentiation. Moreover, the capacity of anti-PD1 to elicit interferon-gamma production by tumor-infiltrating T cells was reduced upon social stress [108]. These data indicate that emotional distress in younger patients may potentially influence the anti-PD1 treatment outcome. Hence, a combination of stress reduction programs along with anti-PD1 immunotherapy might benefit young patients. However, so far there are no clinical studies that could prove the efficacy of such treatment interventions. Therefore, further studies should be designed, especially in young patients, to investigate the benefit of improving mental well-being on the anti-PD1 outcome. 

## 9. Potential Combinational Treatment Options along with Anti-PD1 for Younger Patients

**Figure 2:**
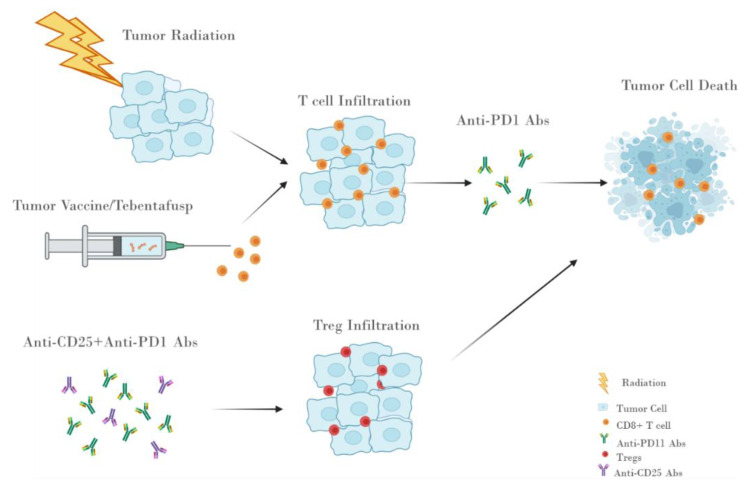
Potential combination treatments to enhance anti-PD1 treatment efficacy in younger patients with metastatic melanoma.

Expanding the benefits of cancer immunotherapy with anti-PD1 or PDL1 to younger patients is probably one of the most urgent challenges in cancer therapy currently. Insufficient tumor-specific priming of cytotoxic CD8+ T cells before anti-PD1 treatment may only result in a dysfunctional state of these cells in the TME. In vitro, PD-1 blockade of unprimed or sub-optimally primed CD8+ cells with tumor antigens induced PD-1+ CD38^hi^ CD8+ cells involved in treatment resistance [109], highlighting the importance to enhance the tumor antigenicity before PD1 blockade. Based on the above evidence of insufficient tumor antigenicity and increased Tregs, we propose that in combination with immune microenvironment-enhancing strategies such as anti-PD1 treatment, potential ways to enhance tumor-cell antigenicity may result in maximum clinical benefits for younger patients (Figure 2). 

### 9.1. Radio Therapy

Radiotherapy (RT) is regularly used to treat cancer and is known to enhance the cross-presentation of tumor antigens in the draining lymph node and directs T cell infiltration into the tumors [110,111]. The ability of radiotherapy to induce potential tumor antigen-specific immune response provides a strong rationale for combining radiation and anti-PD1 treatment. Accordingly, several clinical trials in different tumor types proved that radiation may increase the anti-cancer treatment effect of immune checkpoint inhibitors [112,113,114,115,116,117] and that radiation can be safely combined with anti-PD1 treatment. Besides, combination treatment with radiation and anti-PDL1 significantly reduced the infiltration of tumor-associated MDSCs, and Treg cells in the TME, resulting in more significant tumor regression [114]. In addition, the sequence of combination treatment is also crucial for significant clinical benefits. The radiation before anti-PD1 treatment is likely to open up tumor cell content to immune cells, leading to immune activation and eventually exhaustion, which can be reversed using anti-PD1 treatment. Hence, irradiating tumors before anti-PD1 treatment when applicable may result in better clinical responses in younger patients by enhancing tumor antigenicity.

### 9.2. Vaccination and TCR-Based Therapies

Vaccination with tumor-associated antigens or neoantigens might be an option to induce a T cell response that can then be boosted by PD-1 inhibition. One of the most frequently overexpressed intracellular proteins in melanoma is gp100 [118]. Hence, vaccination with gp100 peptides or RNA may offer a potential strategy to combine with anti-PD1 treatment in younger patients. Short-lived gp100 vaccination primes tumor-specific CD8+ T cells, redirects T cell accumulation in tumors, and induces superior anti-tumor activity along with memory formation [119]. Besides, insufficient priming with gp100 antigen before anti-PD1 treatment resulted in a dysfunctional state of CD8+ T cells and treatment resistance in a melanoma mice model. However, this state of CD8+ T cells can be reversed by optimal priming with gp100 vaccination [109]. Similar to radiotherapy, this study further revealed that sequencing gp100 vaccination before anti-PD-1 treatment is vital for therapeutic success [109]. In addition, for the treatment of metastasized uveal melanoma—a tumor which shares some homologies with cutaneous melanoma in young patients such as being poorly immunogenic—treatment with tebentafusp, a bispecific fusion protein comprising an engineered TCR- to recognize the gp100 peptide presented on HLA-A*02:01 with high affinity, displayed safe and promising clinical activity [120]. Once bound to cancer cells, tebentafusp recruits a broad range of T cells regardless of their natural TCR specificity into the tumor, thereby activating selective killing of gp100 expressing cancer cells. In cutaneous melanoma, tebentafusp is tested in combination with PD-L1 and/or CTLA-4 blockage (NCT02535078). 

Other vaccination strategies which aim to target the immunosuppressive TME have been proposed. For example, a new peptide vaccine called IO102-IO103, which consists of Indoleamine 2, 3-dioxygenase (IDO) plus PDL1 peptides. First results in a phase I/II trial revealed an overall response rate of 79%, with 45% complete responses in melanoma patients when combined with the PD-1 antibody nivolumab [121]. In addition, upon treatment, vaccine-specific T cell responses in the peripheral blood and T cell influx at the TME were observed. Similarly, a dendritic cell vaccine targeting FOXP3 exhibited a substantial anti-tumor effect by increasing cytotoxic T cell response and reducing the percentages of Tregs in TME in a melanoma mouse model [122,123]. These observations strongly suggest that anti-PD-1-mediated resistance can potentially be prevented by concomitant vaccination therapies that drive immune infiltration into the TME, which are safe and efficient. Hence, more studies are required to develop combination treatment with vaccine-based therapies and anti-PD1 treatment. 

### 9.3. Treg Antibody-Based Therapies

From the current outlook, anti-PD-1 may have to evolve with novel Treg depletion strategies or therapies that reduce Treg function for better results and minimal shortfalls. For example, CD25 is highly expressed in FOXP3+ Treg cells but not as much on CD8+ T cells. Therefore, bispecific antibodies that recognize CD25 and PD-1 can be designed for the depletion of PD-1+ Tregs. This minimizes their presence and conditions the tumor for PD-1 blockade on PD-1+ effector T-cells rather than Tregs. Accordingly, a combination of anti-CD25 mAb with an anti-PDL1 mAb induced tumor regression in 90% of animals, delaying tumor progression and significantly increasing overall survival in the mice model [124]. Furthermore, in vitro treatment with RG6292 (anti-CD25 mAb) selectively depleted Treg cells without affecting IL-2 signaling in CD8+ T cells [124]. Evidently, compared to ipilimumab, a mAb that targets CTLA4, which is also highly expressed in Treg cells, tumors treated with anti-CD25 (RG6292) showed a higher CD8+/Treg ratio and activation of infiltrating CD8+ T cells. Hence, bispecific antibodies that recognize CD25 and PD-1 may effectively deplete PD-1+ Tregs and condition the tumor for PD-1 blockade on PD-1+ effector T-cells rather than Tregs. A phase II clinical trial of RG6292 in patients with solid tumors is currently recruiting an entry-into-human study to evaluate the safety and identify the dose (NCT04158583).

## 10. Conclusions and Future Perspectives

In conclusion, cancer immunotherapy with anti-PD1 or PDL1 is certainly a dormant method to reverse the exhaustion and to enhance the antitumor immune responses of pre-existing tumor-specific T cells. Evolving data suggests that younger patients are more likely to develop rapid disease progression than elderly patients with metastatic melanoma. Both tumor intrinsic (ex: reduced tumor antigenicity, Treg infiltration) and extrinsic features (ex: female gender hormones, psychological stress, gut microbiome) may be responsible for such reduced efficacy for anti-PD1 treatment in younger patients. Hence, age-related differences in immune response should not be neglected in anti-PD1 immunotherapy design and analysis, and should be kept in mind for therapeutical stratification of patients. Potential combinational treatments should be further investigated to improve the anti-PD1 efficacy in these patients. Anti-PD1 treatment in combination with tumor antigen enhancing strategies such as radiation, vaccination, and Tregs depletion strategies may be more suitable for younger patients. 

Furthermore, stress reduction programs with established social support networks and psychological therapy can also be utilized as a complementary approach. A combination of these strategies along with immunotherapies could significantly benefit patients under emotional stress and decrease the harmful consequences of neuroendocrine disruption. In addition, a comprehensive understanding of the gut microbiome in young patients may facilitate a simple and supple solution to overcome resistance to anti-PD1 treatment in these patients. Probiotics, non-absorbable oligosaccharides, or a fecal transplant from healthy donors are potential interventional strategies in young patients with gut microbiome dysbiosis. However, we are far from that, and further research is warranted for any conclusions and therapeutical interventions.

Finally, this review is based on both prospective and retrospective clinical studies that show an association with age and anti-PD1 treatment outcomes in melanoma patients. It must be noted that each study has its own limitations, especially the randomized nature of the prospective studies and the selection bias associated with retrospective studies (ex: younger patients may have worse prognostic characteristics: LDH, Braf mutation, tumor stage, origin of primary melanoma) that may have a certain influence on the findings. Therefore, a large-scale meta-analysis including all potential confounders with different age cut-offs at an individual level should be performed further to validate the impact of age on anti-PD-1 treatment. In addition, it is important to note that the influence of age on anti-PD1 treatment outcome may be tumor-specific. A recent meta-analysis revealed that age might have little to no effect on the outcome of NSCLC patients treated with anti-PD1 antibodies [125]. Moreover, the data of age and anti-PD1 response in other tumor types is even more sparse. Hence, the conclusion of this study still cannot be expanded to other tumor types and further studies are required to study the influence of age on anti-PD1 treatment efficacy in other cancer types.

## Figures and Tables

**Table 1:** Summary of clinical studies showing age-related differences in anti-PD1 clinical outcomes in melanoma patients.

Study/Clinical Trial	Anti-PD1 Agent/Study Arms	Age Cut-Off	Response	PFS (HR (95%CI))	OS (HR (95%CI))
Kugel. et al., 2018 [12]	Anti-PD1 (Pembrolizumab)	<62 vs. ≥62	<62 (50%) vs. ≥62 (63%)	-	-
Bastholt. et al., 2019 [13]	Anti-PD1 (Pembrolizumab)	<70 vs. 70–80 vs. ≥80	-	<70 vs. 70–80 (HR: 0.73 (0.58–0.93)); <70 vs. ≥80 (HR: 1.12 (0.79–1.60))	<70 vs. 70–80 (HR: 0.65 (0.48–0.88)) vs. <70 vs. ≥80 (HR: 1.02 (0.66–1.57))
Wu. et al., 2019 [15]	Anti-PD1; Meta-Analysis (3 clinical trials)	<65 vs. ≥65	-	-	<65 (0.74 (95% CI 0.51–0.98)) vs. ≥65 (0.50 (95% CI 0.38–0.62))
Jain. et al., 2020 [14]	Ipilimumab and or Anti-PD1 or Anti-PDL1	<60 vs. ≥60	-	-	<60 (HR: 0.64 (0.57–0.72)) vs. ≥60 (HR: 0.55 (0.50–0.60))
Perier-Muzet. et al., 2018 [16]	Anti-PD1 or Ipilimumab	<65 vs. ≥65	-	<65 (PFS: 3.4 vs. not reached) vs. ≥65 (4.8 vs. 10.1 months)	-
Betof. et al., 2017 [17]	Anti-PD1/PDL1	<50 vs. 50–64 vs. 65–74 vs. ≥75	-	<50 vs. ≥75 (HR: 0.98 (0.55–1.77)); 50–64 vs. ≥75 (HR: 0.82 (0.48–1.41)); 65–74 vs. ≥75 (HR: 0.85 (0.48–1.48))	<50 vs. ≥75 (HR: 0.93 (0.47–1.83)) vs. 50–64 vs. ≥75 (HR: 0.88 (0.47–1.64)) vs. 65–74 vs. ≥75 (HR: 0.83 (0.43–1.60))
Robert. et al., 2015 [11] (CheckMate 066)	Anti-PD1 (Nivolumab)/Dacarbazine	<65 vs. 65–75 vs. ≥75	-	-	<65 (HR: 0.52; CI: 0.32–0.85) vs. 65–75 (HR: 0.44; CI: 0.24–0.81) vs. ≥75 (HR: 0.25; CI: 0.10–0.61)
Weber. et al., 2015 [18] (CheckMate 037)	Anti-PD1 (Nivolumab)/Chemotherapy	<65 vs. ≥65	< 65 (29.3%) vs. ≥65: (36.8%) vs. ≥65–<75 (41.7%) vs. ≥75: (28.6%)	-	-
Robert. et al., 2015 [8] (KeyNote 006)	Anti-PD1 (Pembrolizumab every 2 weeks)/Ipilimumab	<65 vs. ≥65	-	<65 (HR: 0.55 (0.41–0.73)) vs. ≥65 (HR: 0.61 (0.43–0.86))	<65 (HR: 0.65; CI: 0.44–0.95) vs. ≥65 (HR:0.56; CI: 0.36–0.87)
	Anti-PD1 (Pembrolizumab) every 3 weeks/Ipilimumab	<65 vs. ≥65	-	<65 (HR: 0.59 (0.45–0.79)) vs. ≥65 (HR: 0.57 (0.41–0.81))	<65 (HR:0.77; CI: 0.53–1.12) vs. ≥65 (HR: 0.66; CI: 0.44–1.01)
Wolchok. et al., 2017 [19] (CheckMate 067)	Anti-PD1 (Nivolumab)/Ipilimumab	<65 vs. ≥66	-	<65 (HR: 0.58; CI: 0.45–0.73) vs. ≥65 (HR: 0.49; CI: 0.37–0.67)	<65 (HR: 0.62; CI: 0.48–0.81) vs. ≥65 (HR: 0.71; CI: 0.51–0.99)

## Data Availability

Not applicable.
